# Conformational preference of ChaK1 binding peptides: a molecular dynamics study

**DOI:** 10.1186/1757-5036-3-2

**Published:** 2010-01-21

**Authors:** Jiajing Zhang, Christopher A King, Kevin Dalby, Pengyu Ren

**Affiliations:** 1 Department of Biomedical Engineering, The University of Texas at Austin, Austin, TX 78712, USA; 2 Division of Medicinal Chemistry, College of Pharmacy, The University of Texas at Austin, Austin, TX 78712, USA

## Abstract

TRPM7/ChaK1 is a recently discovered atypical protein kinase that has been suggested to selectively phosphorylate the substrate residues located in α-helices. However, the actual structure of kinase-substrate complex has not been determined experimentally and the recognition mechanism remains unknown. In this work we explored possible kinase-substrate binding modes and the likelihood of an α-helix docking interaction, within a kinase active site, using molecular modeling. Specifically kinase ChaK1 and its two peptide substrates were examined; one was an 11-residue segment from the N-terminal domain of annexin-1, a putative endogenous substrate for ChaK1, and the other was an engineered 16-mer peptide substrate determined via peptide library screening. Simulated annealing (SA), replica-exchange molecular dynamics (REMD) and steered molecular dynamics (SMD) simulations were performed on the two peptide substrates and the ChaK1-substrate complex in solution. The simulations indicate that the two substrate peptides are unlikely to bind and react with the ChaK1 kinase in a stable α-helical conformation overall. The key structural elements, sequence motifs, and amino acid residues in the ChaK1 and their possible functions involved in the substrate recognition are discussed.

PACS Codes: 87.15.A-

## 1. Introduction

Protein kinases are a large class of enzymes that catalyze the phosphorylation of proteins [1]. In most cases they deliver a single phosphoryl group from the gamma phosphate of ATP to the hydroxyls of serine, threonine or tyrosine found in protein substrates. The majority of protein kinases are considered to be conventional protein kinases (CPKs), which may be classified as either serine/threonine or tyrosine protein kinases.

The atypical protein kinases (APKs) are a class of protein kinases that lack sequence homology to CPKs. The first two APKs in the so called "alpha-kinases" family [2,3] were myosin heavy chain kinase A (MHCK A) from Dictyostelium [4,5], and elongation factor 2 kinase (eEF-2 kinase [6]). These kinases are involved in the regulation of a wide range of different processes, including protein translation (eEF-2 kinase [7]), myosin association (MHCK [8]), ion channel regulation (TRPM6/ChaK2, TRPM7/ChaK1 [9,10]), and cardiomyocyte differentiation (Midori [11]). Many more APKs of unknown function have been identified in the genomes of a wide variety of different eukaryotes.

As the only kinase in this family for which the three-dimensional structure is available, TRPM7/ChaK1 exists *in vivo *as a fusion between an Mg^2+ ^ion channel domain (referred to as TRPM7) and an APK domain (referred to as ChaK1). Though the APKs display almost no sequence similarity to the CPKs, TRPM7/ChaK1 shows a significant structural resemblance to the CPKs [12]. Crystallographic studies have illustrated the structural features of the ChaK1 dimer [12]. Each of the ChaK1 monomers, composed of 300 residues, folds into a two-lobed structure with the active site located within the cleft between the two lobes. The N-terminal lobe is composed mostly of beta-sheets and contains the Gly-rich loop (^1618^GGGL), which acts as a flexible flap to cover ATP within the active site. The Gly-rich loop is one of the most important motifs in the CPKs and thus it is not surprising to see a similar motif in the APKs. In the C-terminal lobe, the catalytic machinery is built on a beta-sheet platform and is presented to the interlobe cleft. A GxA(G)xxG motif connects this platform to the base of the C-terminal lobe. This C-terminal GxA(G)xxG motif which is highly conserved within the family is also in the equivalent position to the so-called "activation loop" of CPKs. A single peptide chain, referred to as the linker or hinge region, connects two strands in the N- and C-terminal lobe [12].

This family of APKs have been referred to as "alpha-kinases" because their catalytic domain is assumed to have the ability to phosphorylate amino acids located within α-helices [2]. This is quite different from CPKs, which phosphorylate amino acids located within loops, turns or irregular structures [13]. One piece of evidence supporting the notion that the APKs recognize helices is that three MHCK A phosphorylation sites are located within a coiled-coil α-helical region of myosin heavy chains [14]. In addition, the major phosphorylation target for eEF-2 kinase resides within a region that is conserved among all elongation factors, and this region also exists as an α-helix in the crystal structures of EF-Tu [15]. However, the structure of an alpha-kinase in complex with its substrate during the phosphorylation event remains unknown. No direct experimental evidence yet exists to confirm that the kinase substrate is bound as an α-helix during the phosphorylation event.

Although extensive experimental studies have been reported on how protein kinase catalyzes the phosphorylation reaction, the mechanisms are not clearly understood. Considering the experimental challenges, computational modeling based on quantum mechanics/molecular mechanics (QM/MM) methods can help to understand the mechanism at a detailed atomic level of the reaction process. Based on their QM/MM calculations on the catalytic subunit of cAMP-dependent protein kinase, Cheng and McCammon [16] demonstrated that the phosphorylation reaction in their study was mainly dissociative and that the conserved Asp residue serves as the catalytic base to accept the proton delivered by the substrate. The structures of the binding site in the reactant and product states during the phosphorylation event can be illustrated schematically in the study of Valiev et al [17]. For TRPM7/ChaK1, experimental findings [18] suggested that phosphorylation occurs at a conserved serine residue located within the N-terminal α-helical region of the substrate. Owing to the high active site structural similarity between APKs and CPKs, we believe that the terminal carboxyl of Asp residue, the gamma-phosphate of the ATP, and the hydroxyl group of the substrate's serine residue are involved in the phosphorylation-ATPase reaction; therefore, their catalytic contacts are used as loose restrains in our search for peptide conformations.

Molecular simulations based on classical mechanics model can provide useful insights beyond the reach of current experimental techniques. Based on long molecular dynamics simulations of the Abl kinase, Kuriyan and Shaw [19] were able to visualize a characteristic conformational change, the DFG flip, in atomic detail and predicted that protonation of the DFG aspartate controls the flip, in consistent with experimental findings. Adequate sampling of protein configurational space remains a challenge in computational study. Sophisticated simulation algorithms such as replica exchange molecular dynamics (REMD) [20,21] have been successfully applied in probing native structure small proteins with less than 50 residues [22,23]. Alternatively coarse-grained model can be applied to eliminate non-critical degrees of freedom in the system. Reduced potentials have been long utilized for proteins by Levitt and Warshel [24,25] as well as Scheraga [26,27]. Recent development in coarse-grain models is mostly limited to small model compounds [28,29], membranes [30-33], and DNA [34-36]. With molecular modeling, the goal of the current study was to explore the relevant conformations of a substrate peptide bound in the active site of an APK, and to delineate the key residues and structural elements that stabilize the recognition. Our system was comprised of the ChaK1 crystal structure and two peptides representing the kinase's known substrates. Beginning with the initial structures, a number of simulation techniques were used to examine the peptide-kinase interactions, including simulated annealing (SA), steered and replica-exchange molecular dynamics (REMD), and normal mode analysis (NMA). Finally, we reported the key residues and structural elements of the kinases involved in the substrate recognition.

## 2. Computational Approaches

### 2.1. System Setup

#### 2.1.1. The initial structure of ChaK1 alpha-kinase

The atomic coordinates of the TRPM7/ChaK1 kinase domain used in the current work were based upon the published structure in complex with the ATP analogue AMP-PNP (PDB entry 1IA9[12]). This 2.0 Å resolution X-ray crystal structure consists of two homodimeric ChaK1 kinase subunits, but only Chain B was utilized in the current study. The molecule's long, N-terminal dimerization tail structure was eliminated in order to reduce the system's size. Thus the truncated molecule includes residue numbers 1577 to 1828.

Both the ATP and the Zn^2+ ^ion from the original structure were kept. In addition, two Mg^2+ ^ions in the kinase active site are missing in the crystal structure due to crystallization conditions. However, one Mg^2+ ^was reported in the active site of the ChaK1 kinase domain structure in complex with ADP (PDB entry 1IAH[12]). The corresponding site in the ChaK1-AMP-PNP crystal structure (1IA9) is coordinated by a water molecule. This Mg^2+ ^ion was placed into our model via superimposition of the ADP-bound structure (1IAH) superimposed, via alpha carbon RMSD minimization, onto our model.

The placement of the second Mg^2+ ^ion was accomplished by the inspection of the active site Mn^2+ ^ions in the published structure of the CPK cAMP-dependent kinase (PDB entry 1ATP[37]). Due to the extremely high structural homology and conserved residue placement between the APKs and the CPKs, the ion's catalytic geometry was preserved from the CPK to ChaK1. By superposing the two kinases, one of the CPKs Mn^2+ ^ions is shown to coincide with the first Mg^2+ ^ion that had already been placed in the ChaK1 model as described above. Hence the second Mg^2+ ^ion was added to ChaK1 at its equivalent position in the CPK structure.

#### 2.1.2. The substrate peptides

Until now only one endogenous ligand has been proposed for ChaK1: the N-terminal domain of annexin-1 [18]. Specifically, ChaK1 has been shown to phosphorylate Ser5 of annexin-1 in the presence of calcium. In its inactive form, the annexin-1 N-terminal domain exists as an α-helix tucked within the interior of the annexin Ca^2+ ^binding site. However, upon the introduction of Ca^2+ ^ions, annexin undergoes a conformational change that expels the N-terminal helix from the Ca^2+ ^binding site [38]. The crystal structure of annexin-1 in complex with Ca^2+ ^does not include the first 40 N-terminal residues [39]; when it is expelled from the core, the N-terminal domain of active annexin is difficult to crystallize and its helical content is unknown. However, an 11-mer peptide with the same sequence as the annexin N-terminal domain has been crystallized while in complex with the S100C protein [40]. This peptide does exist as an α-helix while complexed to S100C, though whether the peptide exists as a stable helix in solution or undergoes a cooperative transition to an ordered secondary structure upon substrate binding is also unknown. Ser5 of the annexin N-terminal domain has been identified as the sole residue undergoing phosphorylation [18]. A canonical α-helix comprising the 11 residues of the annexin-1 protein existing *in vivo *has been generated. With an acetyl and amide capped for the N and C termini respectively, this ligand substrate (ACE-AMVSAFLKQAW-NH2) is referred to as "annexin" in this work.

In addition, the sequence of a high-affinity 16-mer peptide ligand for the TRPM7/ChaK1 kinase (ACE-RKKYRIVWKSIFRRFL-NH2) has been determined via peptide library screening *in vitro *(Maxim V. Dorovkov et al.: Determinants for substrate phosphorylation by TRPM7 alpha-kinase, unpublished). TRPM7/ChaK1 has been shown to selectively phosphorylate the serine residue of this peptide with high affinity. This ligand was examined as the second ligand substrate in our study, which we referred to as the "engineered peptide".

### 2.2. Molecular Dynamic Simulations

All the molecular dynamics simulations reported here utilized the GROMACS software package [41] and the OPLSAA force field for proteins and peptides [42,43]. Equilibrium bond lengths, angles, torsions, and force constants as well as atomic charges for the ATP molecule were calculated by the PRODRG server [44]. The vdW parameters were transferred from existing OPLSAA model compounds. Two substrate peptides as described were examined respectively in our study. For each peptide, three simulated annealing runs were performed in water molecules only and three simulated annealing runs were carried out with kinase complex soaked with explicit water molecules. In addition, two sets of replica exchange molecular dynamics simulations of the peptides were performed in explicit waters with and without the kinase for comparison purposes.

#### 2.2.1. Simulated Annealing of free peptide in water molecules

For each of the two substrate peptides, three 50-ns independent simulated annealing runs were performed with explicit SPC [45] water only. In each run, the system temperature was first heated from 300 K to 1000 K in 0.8 ns, maintained at 1000 K for 0.2 ns, and then gradually cooled down to 200 K by the default linear cooling schedule in 49 ns. A cubic, SPC [45] explicit solvent water box with 45 Å on each side was generated and periodic boundary conditions were enforced. Cl^- ^counter ions were introduced to neutralize the positive charges of the systems. A 0.9 nm grid neighbor list was updated every 10 steps. Short-range electrostatics and vdW were treated with a 0.9 nm cutoff. Long-range electrostatics were treated via the particle-mesh Ewald algorithm [46,47] with a 0.1 nm Fourier grid spacing and a cubic interpolation order. During the simulation, bond stretching within the proteins was constrained by using the LINCS algorithm [48,49]. The SETTLE algorithm [50] was used to restrain the geometry of the waters. All simulations used the GROMACS MD integrator with a time step of 2 fs. Berendsen temperature coupling [51] was used with a reference temperature of 298 K and with a time constant of 0.1 ps unless otherwise noted.

#### 2.2.2. Simulated Annealing of the Kinase-Peptide Complex

To determine the structure of the substrate peptide when bound to the active site of the kinase, a total of three 50 ns-simulated annealing molecular dynamics simulations were performed for each of the two peptide substrate-kinase complexes. Before annealing, the entire system was energy minimized and equilibration was achieved via steepest descent minimization using a 0.01 nm step and a 100 kJ/mol/nm tolerance. The cell dimension was set to be 74 Å on each side, which can ensure that the peptide and its own image would be separated at least 12 Å. Different thermostats were used for the kinase and the peptide. The peptide was annealed following the same annealing procedure as the free peptide in water as described while the kinase was always kept at room temperature. Other computational parameters were the same as those of free peptide in water unless otherwise noted.

The annealing simulations began with the canonical helix peptide's serine hydroxyl group facing the active site at approximately 2.5 nm distance. As we are only interested in the peptide configuration where the catalytic serine is in the active site with all necessary catalytic contact, two flat-bottomed harmonic distance restraints [52] were introduced. One is between the Ser hydroxyl oxygen atom and the Asp1765 carboxyl carbon atom, with the minimum energy set to distances between 0.3 nm and 0.4 nm; the other between the Ser hydroxyl oxygen atom and the ATP gamma-phosphate phosphorous atom, with the minimum energy set to distances between 0.15 nm and 0.25 nm. The force constant of 1000 kJ/mol/nm^2 ^was applied.

To examine how different starting orientations affect the binding conformation, these simulations started with different initial peptide orientations. Defining the peptide vector as the vector from the C-terminus to the N-terminus in the canonical helix, and the kinase vector as the vector from the ATP alpha-phosphate to the beta-phosphate (across the "cleft" of the active site), simulations were begun with relative peptide-kinase vector angles of 0, 90 and 180 degrees.

#### 2.2.3. Replica Exchange Molecular Dynamics

The Replica Exchange Molecular Dynamics (REMD) method is an enhanced sampling technique for searching the conformational space of peptides and small proteins efficiently [20,21,53]. The REMD algorithm can be summarized as follows: (*i*) A number of replicas of the system (*i *= 1, 2 ...N) are simulated in parallel but at different temperatures *T*_n _(*n *= 1, 2 ...N). (*ii*) During the simulations, a pair of replicas at neighboring temperatures is selected and the exchange is made periodically with the acceptance ratio(1)

where Δ = [*β*_n_-*β*_n+1_] (*E*(*q *^[*i*]^)- *E*(*q *^[*j*]^)). Here *β*_n _and *β*_n+1 _are two reciprocal temperatures, *q *^[*i*] ^is the configuration at *β*_n_, *q *^[*j*] ^is the configuration at *β*_n+1_, and *E*(*q *^[*i*]^) and *E*(*q *^[*j*]^) are potential energies of the systems at these two configurations, respectively.

A total of 217 peptide-kinase complex replicas were used in the REMD simulation. Different temperature gaps from 1.1 K to 2.0 K were used between the neighboring replicas [54]. These gaps were chosen to make sure that the success rates of exchange between replicas are 20% or higher. To generate a set of initial configurations that can broadly cover the conformational space of the complexes, we chose 217 starting configurations randomly from the previous 50-ns annealing MD simulation trajectories. Each replica was subject to 3.0 ns MD simulation with a 1-fs time step and the temperature of each replica ranged from 282.5 K to 602.3 K. The configurations were saved every 2 ps and the exchanges were attempted every 0.8 ps, with the acceptance ratio determined by the Metropolis criterion, which is shown in Eq (1). Position restraints with flat-bottomed potentials were applied in the REMD simulation for all heavy atoms in the kinase and ATP and for all metal atoms with a 200 kJ/mol/nm^2 ^force constant. This was necessary to preserve the structural integrity of the kinase at high temperatures while exploring the conformational space of the peptide substrate. All other simulation details were identical to the annealing protocol unless otherwise noted.

In addition, a 10 ns room temperature MD simulation was set up for each substrate following the REMD. The simulations began after the REMD run with all the artificial restraints removed. All other computational details except thermostat are the same as simulated annealing. The possible interactions among the substrate residues, ATP and ChaK1 residues were analyzed and identified.

REMD simulations of free peptides in water were performed using 217 replicas. The starting configurations were also randomly chosen from the previous peptides in water annealing MD simulation trajectories, and immersed in cubic boxes with 45 Å on each side. The simulation protocols used was the same as those for peptides binding with kinase.

#### 2.2.4. Steered Molecular Dynamics Simulations

Three 30 ns room-temperature molecular dynamics simulations were then performed to steer each peptide substrate. The same distance restraints with force constants of 1000 kJ/mol/nm^2 ^were used to pull the substrates into the active site from 25 Å away. The substrates were forced to remain as α-helices during the steering MD by restraining hydrogen bonds. Specifically, a set of distance restraints with force constants of 2000 kJ/mol/nm^2 ^were applied between the oxygen atoms in the C=O group of an amino acid *i *and the nitrogen atoms in the N-H group of the amino acid *i*+4 in the substrate.

## 3. Results and Discussion

### Conformational propensities

From the REMD simulation, we have investigated the conformational distribution of the two peptide substrates, free in water as well as bound to kinase. REMD trajectories derived from all 217 replicas were analyzed using the temperature weighted histogram analysis method (T-WHAM) [55,56] to evaluate the conformational preference of each residue at room temperature. The use of high temperature in REMD is to facilitate the sampling of conformation space near the room temperature. We first calculated the conformational population distribution with respect to the backbone dihedral angles for each residue in water or bound to kinase. Note that the conformational free energy can be calculated from the population *P *as -*RT*ln*P*. Secondly, we also evaluated the occurrence of a segment of the peptide as a stable α-helical structure by examining the secondary structure profile from our REMD simulation. While the former illustrate the conformational propensity of an individual residue, the latter truly reflects the probability of the peptide existing as a stable helix partially or as a whole.

**Figure 1:**
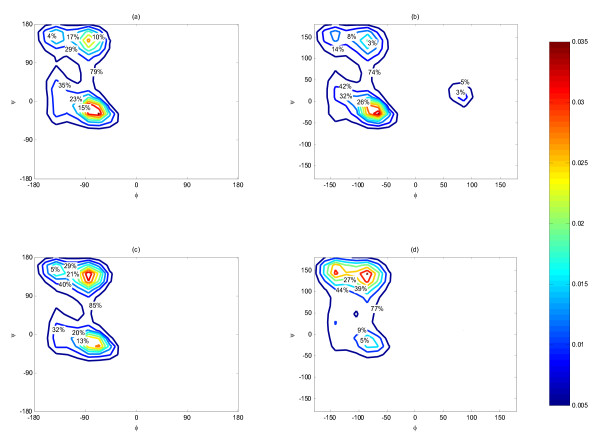
**Conformational population distribution plots of each substrate residue with respect to dihedral angles: (a) when annexin is free in water; (b) when annexin binds with the kinase; (c) when engineered peptide is free in water; (d) when engineered peptide binds with the kinase**. The distribution was calculated from REMD using T-WHAM and normalized. The maps were plotted with contour step levels of 0.5%

The distribution of the peptide backbone Φ and Ψ were sampled from the REMD simulation. As shown in Figure 1a, the overall distribution of annexin (combining all residues) in water showed a strong preference for the α-helix, beta-sheet, and Polyproline II (PPII) regions in the Ramachandran plots. Bins with population less than 0.5% were disregarded in the maps. The overall characteristic is similar to that of the Ramachandran plots obtained from PDB [57] except that the PPII helix region is more populated than beta sheet here. When moving from water into complex, the population around α-helical region (-90° ~ -55°, -50° ~ -10°) increased as shown in Figure 1b compared to Figure 1a, indicating an increased helical preference of about 11% for annexin bound to Chak1. The increase in α-helix was accompanied by the reduction of beta-sheet-like and PPII-like conformations (-140° ~ -60°, +110° ~ +150°).

**Figure 2:**
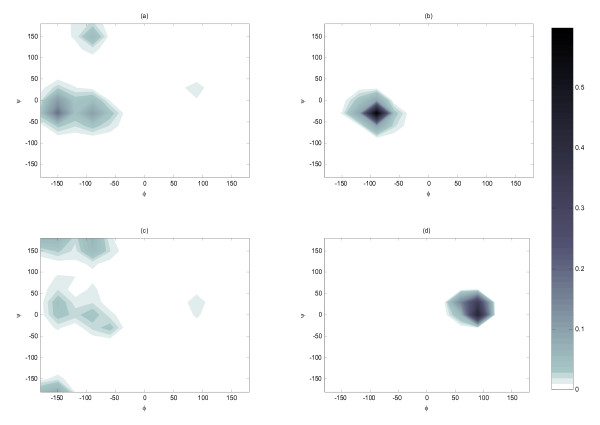
**Conformational populations of individual annexin residue with respect to the backbone dihedral angles: (a) Phe6 when annexin is free in water; (b) Phe6 when annexin binds with the kinase; (c) Ser4 when annexin is free in water; (d) Ser4 when annexin binds with the kinase**. All the distribution was calculated from the REMD room temperature replica and normalized.

The conformational distribution was further decomposed by residue. The majority of annexin residues, i.e. Ala1, Met2, Val3, Phe6, Lys8, Gln9, and Ala10, had much higher propensities to exhibit α-helical conformation when the substrate was bound to Chak1 than when free in water. For illustration, we presented the comparison of Phe6 population distributions in Figure 2a (annexin in water) and Figure 2b (annexin bound to ChaK1). Notably, shown in Figure 2c and Figure 2d, Ser4 residue was responsible for the emerging left-handed α-helical preferences when binding to kinase. Exclusion of the Ser4 did not affect the other population distribution except the left handed helical population in the bound state diminished. The results for other residues were shown in [Additional file 1]. To ensure we were not observing the artifacts of the flat-bottomed restraint applied between the Ser and kinase, we removed the distance restrains and performed 10 ns room-temperature MD simulation starting with the final structure from REMD simulations. The conformational distribution per residue from this simulation was plotted and shown in the [Additional file 1]. We found that all the residues of the annexin except for Ala10 displayed essentially the same conformational preference with and without distance restraints. Ala10 showed stronger preference for α-helix in the REMD simulation.

**Figure 3:**
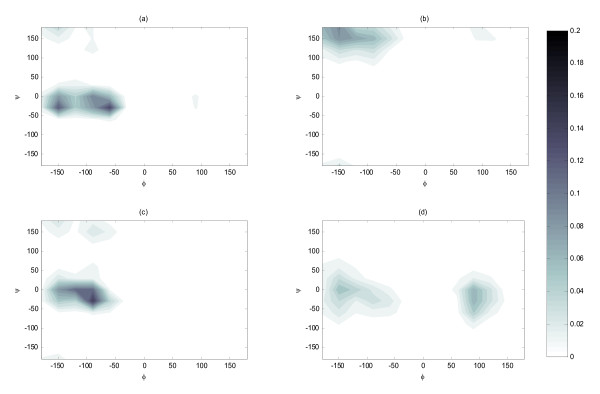
**Conformational populations of individual engineered peptide residue with respect to the backbone dihedral angles: (a) Phe12 when engineered peptide is free in water; (b) Phe12 when engineered peptide binds with the kinase; (c) Ser10 when engineered peptide is free in water; (d) Ser10 when engineered peptide binds with the kinase**. All the distribution was calculated from the REMD room temperature replica and normalized.

Interestingly the engineered peptide behaved differently from annexin. The overall propensity for α-helix dropped by 10% moving from water (Figure 1c) to Chak1 (Figure 1d). In addition, when examining the individual residues, we found that only about half of the residues (Arg1, Trp8, Lys9, Ser10, Phe12, Arg14, Phe15, and Leu16), exhibited a pronounced α-helical population (or a global minimum in the free energy map) when free in water (see [Additional file 1]). Among them, only Arg1 and Leu16 showed slightly higher or equal propensity for α-helical conformation after binding the kinase. The rest exhibited a weaker propensity for the α-helical conformation after binding to the kinase. As an example, the conformational distributions of Phe12 were contrasted in Figure 3a and Figure 3b. In addition, Ser10 was responsible for the appearance of left-handed α-helical population in bound state (Figure 3c and Figure 3d). The other half of residues showed no or little α-helical conformation in water, and among them only Lys2 and Lys3 showed an increased propensity for α-helical conformations in bound state. We also compared the conformational distribution with and without distance restraints in a similar manner to the annexin study (above). The overall distribution averaged over all residues was not affected by the removal of the restraint. Nonetheless, most of the individual residue seemed to have sampled different regions of conformational space (See [Additional file 1]). These results indicated that when in complex with the Chak1 the engineered peptide behaved significantly more like a random coil than annexin.

**Figure 4:**
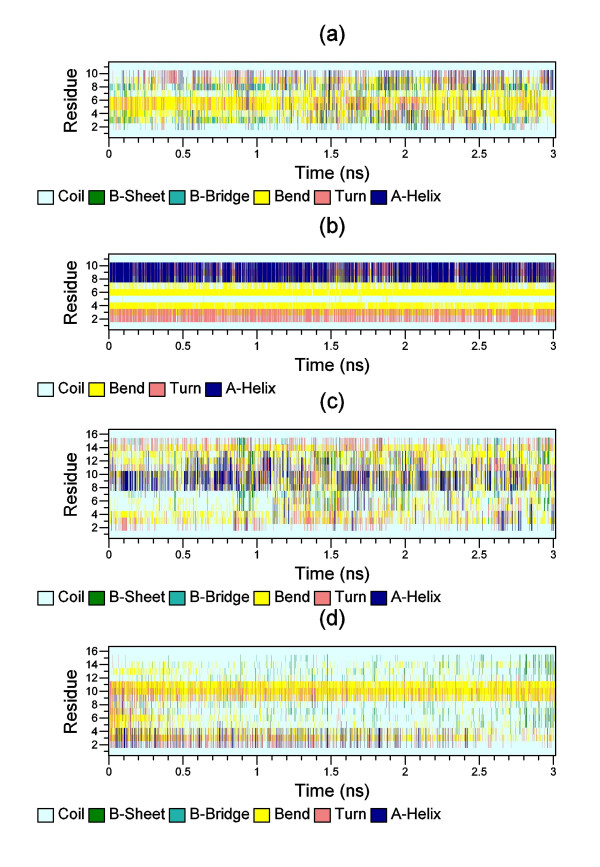
**Peptide secondary structures as function of time calculated by DSSP program **[59]**for (a) annexin is free in water; (b) annexin bound to kinase; (c) engineered peptide is free in water; (d) engineered peptide bound to kinase**.

In addition to the Ramachandran plots of residues, we also examined the secondary structure profile of the whole peptide from our REMD simulation. In the case of annexin in water, few short-lived α-helices appeared in different positions of the annexin peptide in Figure 4a. However, when annexin was bound to kinase (Figure 4b), a segment from residue 7 to 11 near the C-terminal of annexin existed persistently the helical structure, while the remainder of the peptide adopted stable turn, coil and bend structures throughout the whole simulation. In the case of engineered peptide in water (Figure 4c), a small α-helix structure (residues 8-12) was formed and appeared for quite some time, and a few other short-lived alpha helices appeared in different positions. Nonetheless, when the engineered peptide was bound to kinase (Figure 4d), the small alpha helix (residues 8-12) disappeared and only one of the few short-lived α-helices (residues 2-4) appeared. Hence, the engineered peptide favored random coil when binding with kinase. All these findings were in good agreement with the Ramachandran plot observations.

### Global search for stable structures

**Figure 5:**
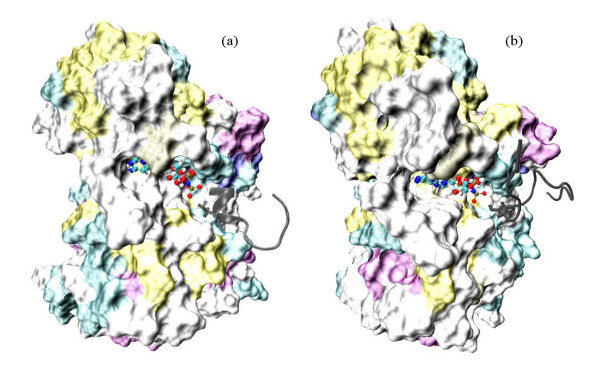
**The low energy structures obtained from simulated annealing simulations for the kinase in complex with (a) annexin; and (b) engineered peptide**. The Ser residue is in stick and the ATP molecule is in ball-and-stick representation. The kinase is in surface representation and both the substrates are in dark gray. Figures were generated using the VMD program [60].

As alternatives to REMD, several simulated annealing runs were applied to search for the minima energy structures of each substrate peptide. REMD involves simulations of multiple replicas at increasingly higher temperatures and the enhanced sampling is achieved via swapping configurations between neighboring replicas. In simulated annealing, the peptide was quickly heated to high temperature so that it can sample rare high-energy states; the slow cooling allowed the peptide to find low energy configurations. For the annexin peptide in water, most simulated annealing runs showed that the annexin folds into a structure which consisted of a random coil and a helix turn. In the case of engineered peptide free in water, simulations showed that the minimum-energy conformations were mixtures of loops and random coils. When annexin was bound to kinase, despite their dramatically different starting orientations, the local minimum structure with one and a half helical turn around the Ser4 residue was observed (Figure 5a) from one of the three simulations; and the other annealing simulation finds a minimum structure with one helical turn around C-terminal. The third annealing simulation did not show any helical peptide structure. For the engineered peptide bound to kinase, no apparent helical structure (Figure 5b) was ever observed in the three simulations. Overall, the structures observed for both peptides in the REMD simulations were similar to those obtained from simulated annealing.

We further compared the ChaK1-annexin complex structures before and after the 50 ns-simulated annealing to inspect the kinase structural change in response to the substrate binding. Since the thermostat was set at room temperature for the ChaK1, most of the kinase residues remained stably packed during the simulations. However, relatively large structural displacements between the structures were found in a small α-helix (Res 1648-1659) and two other regions, Gly-rich loop (Res 1618-1622) and C-terminal GxA(G)xxG motif (Res1792-1797). Firstly, the Gly-rich loop shifted slightly downwards and outwards from the ATP-binding site, which appeared to cover ATP more fully. The Cα of Gly1619 shifted about 3 Å from the original structure. Secondly, the small α-helix (Res 1648-1659) located near the active site appeared to be twisted and shifted a distance away from the original helix. As a result, the Cα of Pro1650 was shifted about 4 Å away, and the delta Nitrogen atom of Asn1654 was shifted by 7 Å as well. Thirdly, after the simulation, the structure of the conserved C-terminal GxA(G)xxG motif (Res1792-1797) was quite different from the one before. This motif is located in the so-called "activation loop" region in the conventional protein kinases. In many cases of CPKs, after experiencing a conformational change induced by phosphorylation, the activation loop presents a beta strand for pairing with the peptide substrate. Here in the case of the APK, the alpha carbons of residue Leu1796 and Gly1797 shifted over 3 Å. The observed flexibility may be partly due to the presence of several glycine residues in this region, and partly because this loop may actually be involved in the substrate recognition and binding.

### Normal mode analysis of the APK structures

The Chak1 flexibility is important in understanding whether the kinase can adapt its conformation to substrate binding. Normal mode analysis is a powerful tool that can be used for the analysis of collective motions in proteins that are beyond the time scales of regular MD simulations. We performed a coarse-grained normal mode analysis [58] to determine the capability of the ChaK1 further relaxing to accommodate the peptide substrate. The coarse-grained NMA clearly indicated a "breathing" motion between the C and N terminal lobes that would open up the active site. The similar motion was indeed observed the SA simulation, suggesting that the conformational flexibility of ChaK1 have been sampled effectively in our simulated annealing runs.

### Coupled folding and binding of the substrate peptides

**Figure 6:**
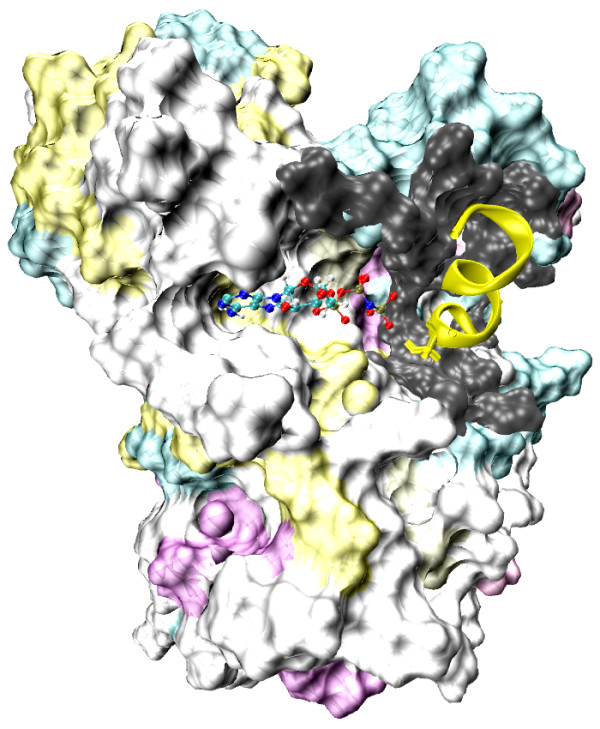
**The structure of ChaK1 binding with annexin from the room temperature MD simulation**. The ATP molecule is in ball-and-stick representation. The kinase is in surface representation and the annexin peptide is restrained into stable α-helix while binding. The kinase atoms that are within 3 Å of the annexin atoms are in surface representation and colored in dark gray. Figures were generated using the VMD program [60].

Suppose the substrates could fold into stable α-helical structures in complex with alpha-kinase, the interesting question arises as to whether the substrate folds into a helical structure and then consequently binds with the kinase, or whether it first unfolds itself to be a disordered peptide after which the binding induces its folding. Based on our simulated annealing runs, the peptides were observed to become disordered when bound compared to the initial ideal helical structure. Nevertheless, we also examined the possibility that the kinase may bind the substrates that have already folded as stable helices. A 30 ns-room-temperature steered molecular dynamics simulations were performed for each substrate starting from different orientations. The substrates were pulled into the active site using the distance restraints between the Ser hydroxyl oxygen and the Asp1765 carboxyl carbon atom, and between the Ser hydroxyl oxygen and the ATP gamma-phosphate phosphorous atom. In the meantime, they were maintained as α-helices at the same time via hydrogen bond restrains. The final structure was illustrated in Figure 6. In this case, it appeared that the substrate either cannot gain any access or had very restricted access to the active site, as it clashed with surrounding loops and strands near the active site of the ChaK1. The kinase was clearly unable to accommodate to the helical substrate. Measured from the resulting structure, the distance between Ser residue of the substrates and gamma-phosphate of ATP was approximately 9 Å at the closest, far beyond the reach of catalytic residues.

### Key roles of conserved residues in structure stability and substrate recognition

For the room temperature conventional MD simulations following the REMD without the distance restraints between peptide and ChaK1, the 10 ns trajectories were used to analyze each substrate. From the simulations, the backbone root mean square deviations (RMSD) of ChaK1 from crystal structure were computed to be ~ 2.4 Å, a typical value for MD simulations of proteins.

Critical contacts between the peptide substrates and ChaK1 identified from the simulation trajectories were shown in Table 1 and Table 2. Research findings based on the X-ray crystal structure reported that the Gly-rich loop forms H-bonds with beta and gamma-P groups of ATP [3]. As shown in Table 1, in our simulations, the Gly-rich loop forms H-bonding networks with the ATP and with the Ser residue at a very close proximity. Indeed we observed that the Gly-rich loop shifted downwards and outwards from the binding pocket during the simulated annealing to facilitate such interactions.

**Table 1:** The mean contact distance between residues in ChaK1 (first column) and annexin (first row).

	*Met2*	*Val3*	*Ser4*	*Ala5*	*Phe6*	*Lys8*	*Trp11*	*ATP*
*Gly1619*				0.84(33.1%)	0.69(93.6%)			0.65(100%)
*Gly1620*			0.63(98.9%)	0.58(99.9%)	0.53(100%)			0.62(100%)
*Leu1621*			**0.61(100%)**	0.66(97.3%)	0.66(97.3%)			**0.79(67.5%)**
*Arg1622*								**0.53(100%)**
*Lys1646*								***0.68(100%)***
*Glu1651*					0.92(24.36%)	***0.92(31%)***		
*Glu1718*								***0.87(20%)***
*Lys1727*		**0.74(83.7%)**	**0.79(55.8%)**					
*Asn1730*	0.64(100%)	0.60(100%)	0.86(12.3%)					
*Asn1731*	**0.44(100%)**	0.71(99.9%)	0.85(7.7%)					
*Asn1732*	**0.55(100%)**	0.83(34.1%)						
*Asp1765*			0.77(86.6%)					
*Gln1767*			**0.77(90.9%)**					
*Ala1794*	0.83(20%)							
*Asn1795*	0.47(100%)						**1.0(10.1%)**	
*Leu1796*	0.77(72.1%)							
*Gly1797*	0.79(60%)							

The contact was considered only when the distance between center mass of residues was shorter than 1.0 nm. The probability of occurrence of the interaction was calculated and shown in the parenthesis. The numbers highlighted in bold are identified as hydrogen bonding interactions and italic labels are salt bridges.

**Table 2:** The mean contact distance between residues in ChaK1 (first column) and engineered peptide (first row).

	*Lys2*	*Arg5*	*Ile6*	*Lys9*	*Ser10*	*Ile11*	*Phe12*	*Arg13*	*ATP*
*Gly1618*									**0.80(100%)**
*Gly1620*		**0.89(68.1%)**	0.94(76.8%)		0.97(59.4%)				0.66(100%)
*Leu1621*		0.89(68.6%)	0.69(100%)		0.73(99.6%)	0.90(78.7%)			**0.81(100%)**
*Arg1622*									**0.68(100%)**
*Lys1646*									**0.76(100%)**
*Leu1649*		0.85(70.6%)	0.87(97.5%)						
*Glu1651*	**0.95(72.8%)**	***0.76(93.0%)***	0.67(100%)						
*Val1652*			0.72(99.4%)		1.0(48.5%)	0.91(74%)			
*Gly1655*			0.82(91.7%)		1.0(54.4%)	0.95(59.2%)			
*Glu1718*									**0.91(100%)**
*Lys1727*				**0.84(80.7%)**					**0.90(99.9%)**
*Asn1731*				1.0(57.5%)	0.77(98.4%)	0.73(92.1%)			
*Asn1732*				0.96(67.9%)	0.92(73.1%)	0.90(82.1%)	0.98(64.1%)	**0.74(100%)**	
*Asp1734*				***0.93(64.7%)***				***0.95(65.7%)***	
*Asp1765*					0.97(70.9%)				
*Asn1795*						0.67(97.3%)	0.74(95.4%)		
*Asp1799*						0.92(70.7%)	0.77(99.4%)	***0.6(100%)***	
*Ala1800*					0.89(83.5%)	0.81(87.5%)	0.78(92.9%)	0.90(97.0%)	

The contact was considered only when the distance between center mass of residues was shorter than 1.0 nm. The probability of occurrence of the interaction was calculated and shown in the parenthesis. The numbers highlighted in bold are identified as hydrogen bonding interactions and italic labels are salt bridges.

Between the Gly-rich loop and the small α-helix (Res 1648-1659), there is a strand that contains an important conserved Lys1646. During our simulation the Lys1646 formed a salt-bridge with the Glu1718 and forms H-bonding with the ATP, which was suggested previously by experimental study [3,12]. In typical CPK, there is a loop between two small, hydrophobic strands, which is known as the 'catalytic loop' as the conserved aspartate is intimately involved in catalysis. However, this catalytic loop is absent from the ChaK1 and so is the conserved lysine. Therefore, it was speculated that in ChaK1 another lysine, Lys1727, may play the same role as PKA's Lys168 does from the catalytic loop [12]. We found that Lys1727 formed a salt-bridge with the catalytic Asp1765 and H-bonds with the substrates during the simulation. Specifically, Lys1727 forms H-bonds with Val3 and Ser4 from the annexin substrate and Lys9 from the engineered peptide. Our simulation results confirm this experimental speculation about the role of Lys1727.

In addition, as we discussed the conserved C-terminal GxA(G)xxG motif (Res1792-1797) is typical to be involved in peptide substrate recognition, we found that Asp1795 interacts with the Met2 from annexin via H-bonds and that Asp1799 forms a salt-bridge with Arg13 from the engineered peptide. These findings indicate that this motif is involved in peptide substrate recognition.

Additional specific interactions observed in our simulation are listed in Table 1 and Table 2, and some of those are not reported in the experiments. We believe that those interactions are important for substrate binding.

## 4. Conclusion

We carried out extensive explicit solvent MD simulations for ChaK1 binding with two substrates to investigate the substrate conformational preference upon binding. REMD simulations of 0.65 microseconds total was employed and the conformational free energy of peptides were analyzed using T-WHAM. The φ-ψ population maps revealed that the individual residues in the engineered peptide had little helical propensity in the bound state. In addition, the engineered peptide did not show any stable α-helical fragments when bound to ChaK1 during the REMD simulation or simulated annealing. However, in the case of annexin substrate, most of the residues displayed significant α-helical inclination in bound and solution states. Each individual annexin residue appeared to have a high α-helical probability of existing as a helical conformation. Moreover, both REMD and SA simulations suggested that the annexin can display a stable helical segment with one to two turns when bound to ChaK1. The steered molecular dynamics simulation, where the two peptide substrates, while maintained as helices, were slowly pulled toward the active site further confirms that it is unlikely that the substrate is unable to dock into the active site as a helix presenting the peptide serine residue for phosphorylation.

Computational sampling remains a challenging task even with technique such as replica-exchange molecular dynamics that provided 0.65 microseconds simulations of dynamics. Especially when bound with protein, the free energy landscape of peptide substrate is likely to have very steep wells that trap the system into local minima.

Our coarse-grained normal mode analysis of ChaK1 indicated that ChaK1 conformational fluctuation has been sufficiently sampled such that it is unlikely we are missing configuration where the ChaK1 undergoes substantial conformational change to adapt to the substrate binding. We did observe that upon binding, the Gly-rich loop, a small α-helix and C-terminal GxA(G)xxG motif displayed high degrees of flexibility; some of the amino acid residues in these regions moved as far as 7 Å with respect to their positions in the initial crystal structure. These regions are likely in the substrate recognition and binding. Several key interactions identified from the MD simulations are in agreement with those proposed in the previous experimental study. The residues identified in these interactions, highly conserved in APK kinase family that ChaK1 belongs to, are believed to play important roles in catalytic function [3,12].

In summary, this computational study suggests that the peptide substrate is not required to be a stable helix in order to bind to ChaK1 for phosphorylation. In fact, out of the two peptide substrates we investigated, only one displayed random helical segments (1-2 helical turns) according to the simulations.

## Supplementary Material

Additional file 1 This file contains conformational population distributions of individual annexin/engineered peptide residue with respect to dihedral angles, when the annexin/engineered peptide is free in water or binds with kinase. Click here for file
