# Supplementary material for: Conformational preference of ChaK1 binding peptides: a molecular dynamics study
Source: PMC Biophys. 2010 Jan 21;3:2. doi: 10.1186/1757-5036-3-2 (PMC2831825; doi:10.1186/1757-5036-3-2)
Supplement: Additional file 1 — This file contains conformational population distributions of individual annexin/engineered peptide residue with respect to dihedral angles, when the annexin/engineered peptide is free in water or binds with kinase. [file 1757-5036-3-2-S1.PDF]

Additional file

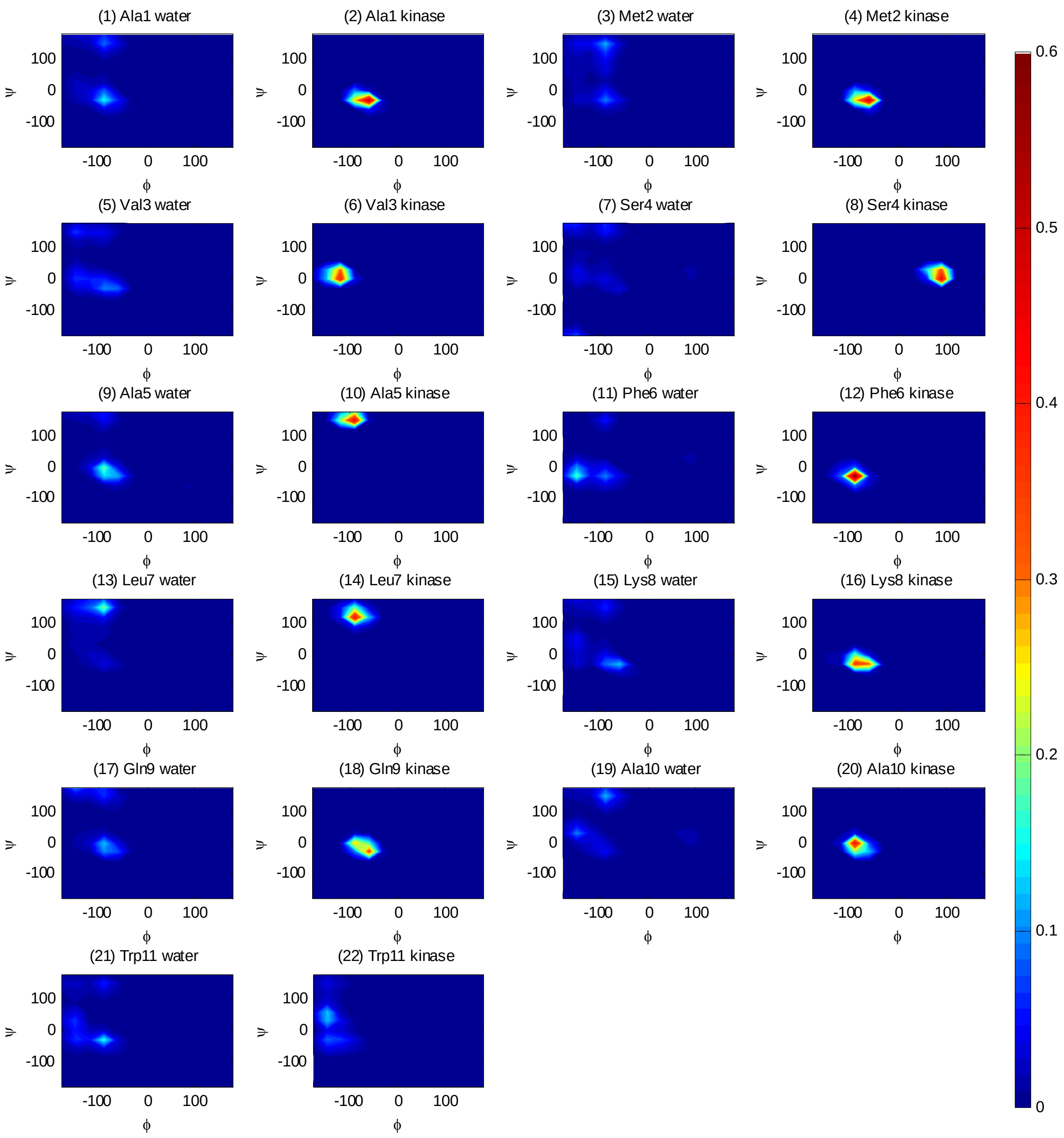

Figure S1. Conformational population distributions of individual annexin residue with respect to dihedral angles. The individual residues are shown in odd numbered panels when the annexin is free in water and in even numbered panels when the annexin binds with the kinase. All the distributions are calculated from the REMD simulation room temperature replica. The distributions are normalized to 1.

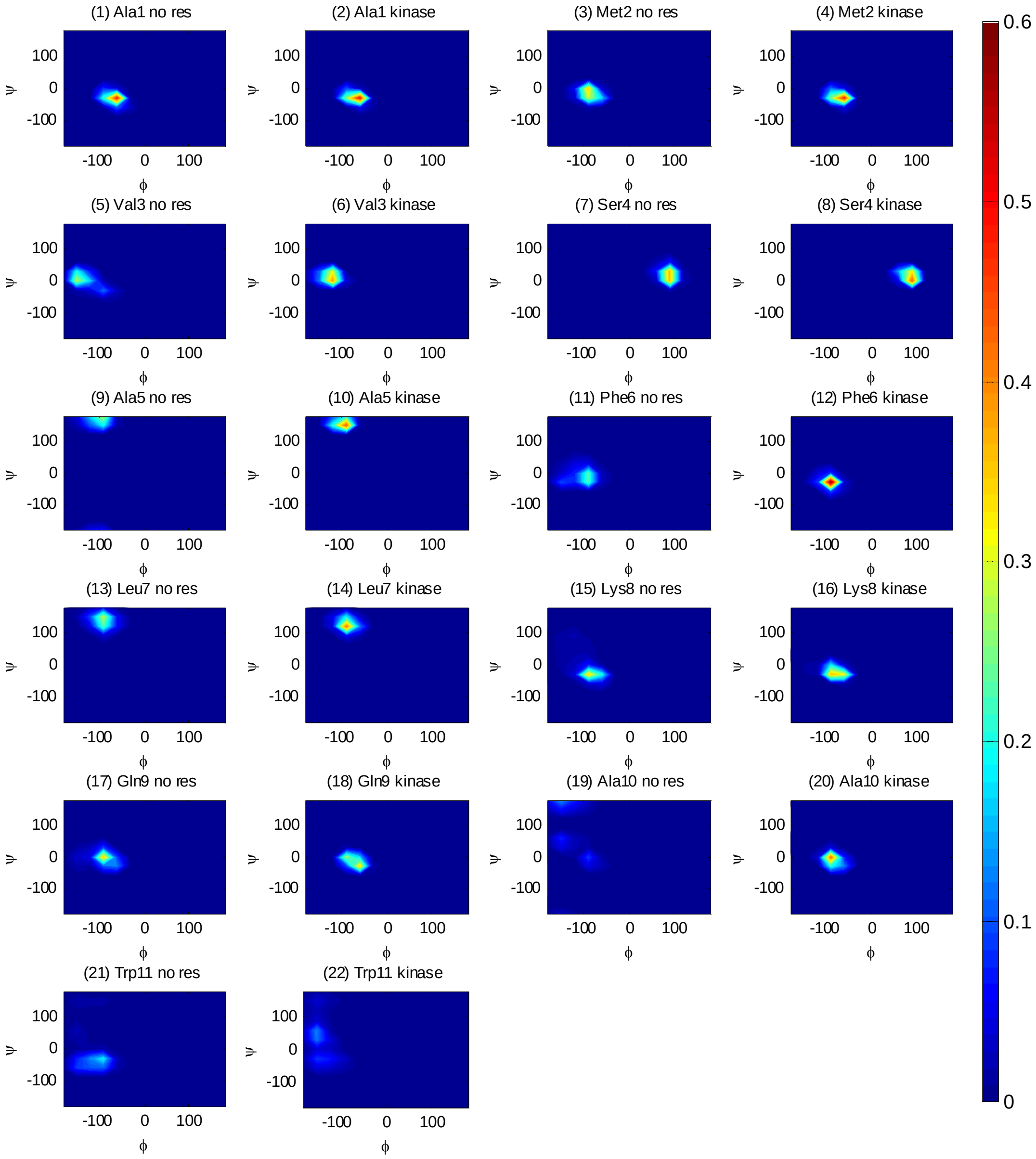

Figure S2. Conformational population distribution of individual residue with respect to dihedral angles when the annexin binds with kinase. The distributions shown in odd numbered panels are from room temperature MD simulation with all the restraints removed and those shown in even numbered panels are calculated from the REMD simulation room temperature replica with restraints enforced. The distributions are normalized to 1.

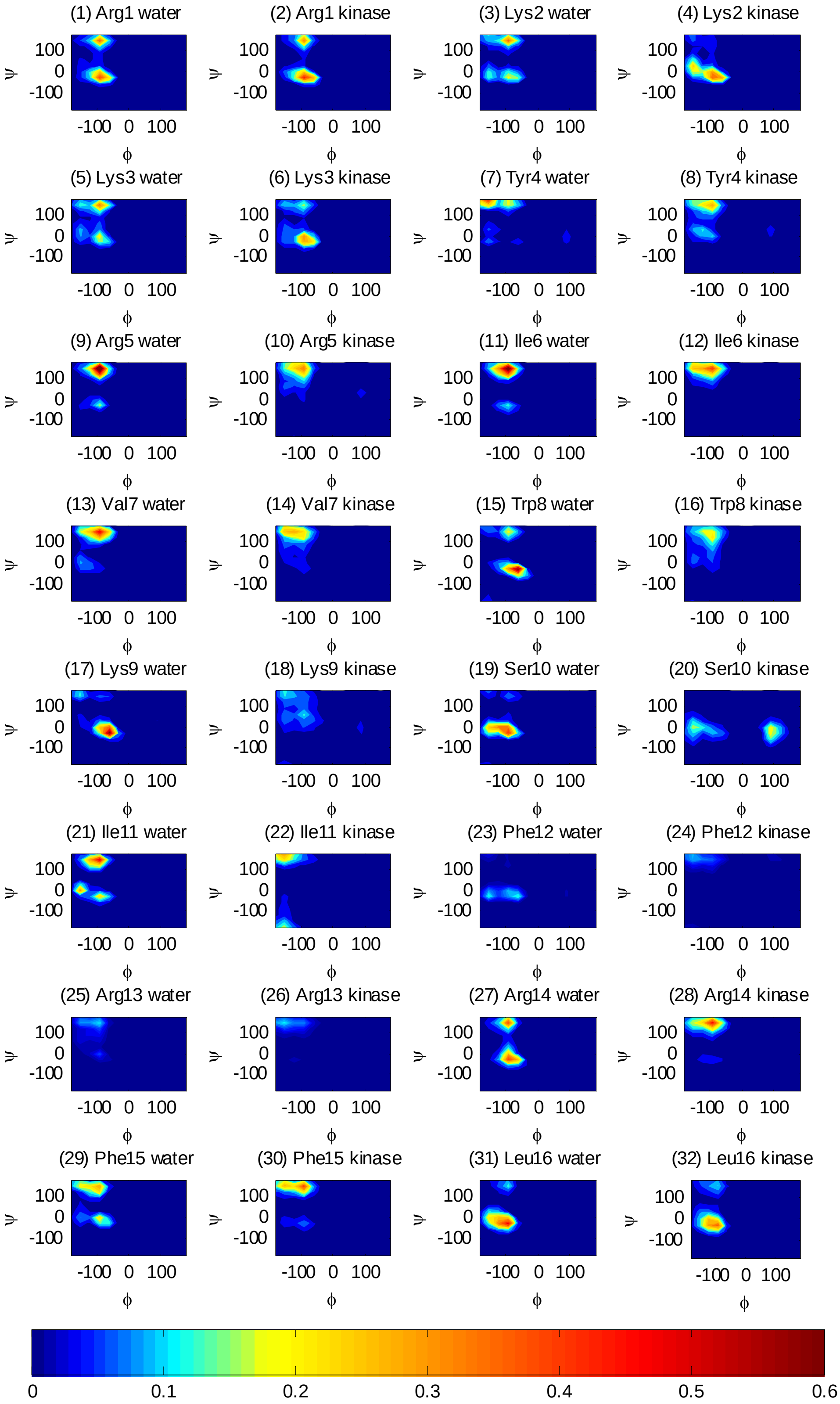

Figure S3. Conformational population distributions of individual engineered peptide residue with respect

to dihedral angles. The individual residues are shown in odd numbered panels when the engineered peptide is free in water and in even numbered panels when the engineered peptide binds with the kinase. All the distributions are calculated from the REMD simulation room temperature replica. The distributions are normalized to 1.

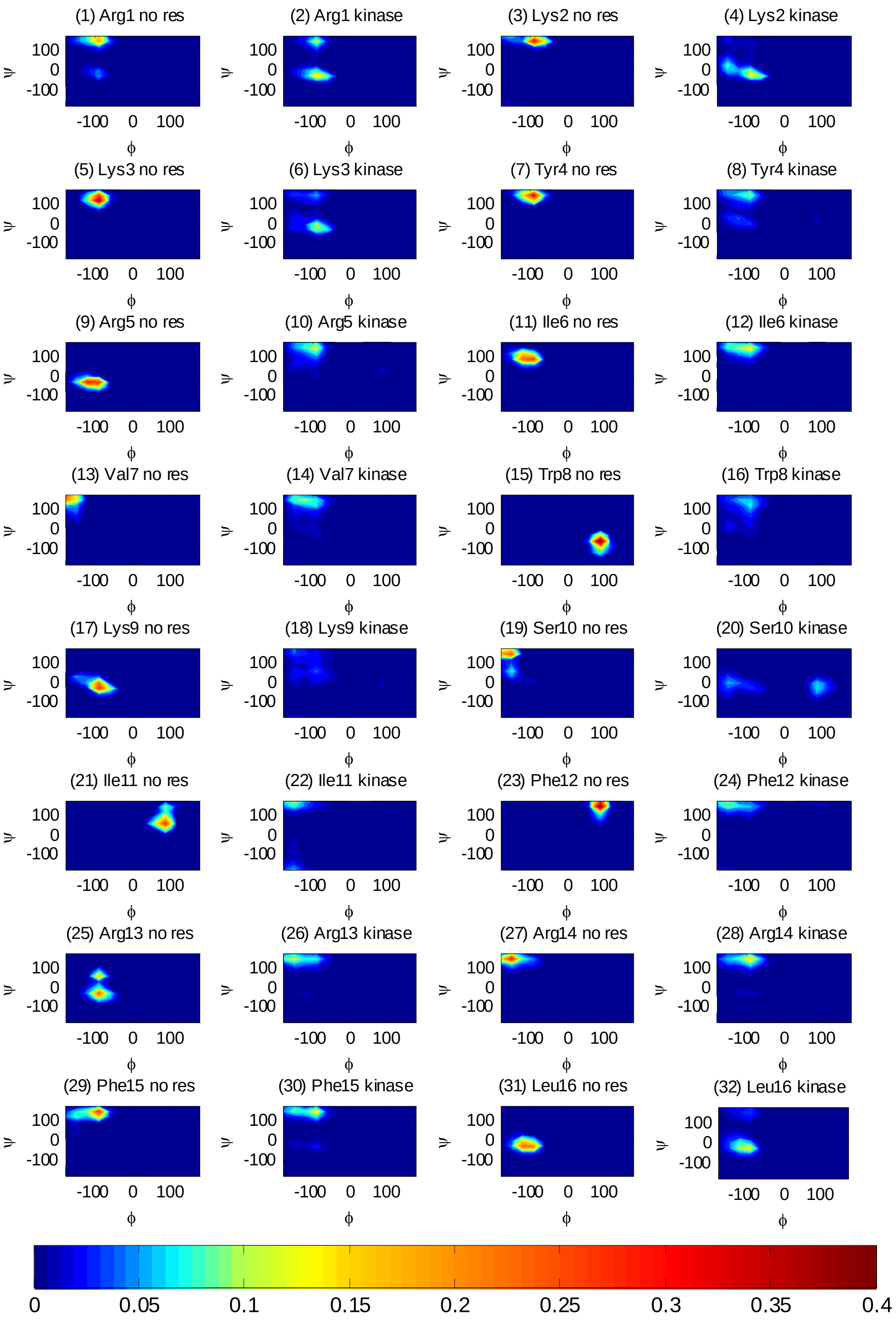

Figure S4. Conformational population distribution of individual residue with respect to dihedral angles

when the engineered peptide binds with kinase. The distributions shown in odd numbered panels are from room temperature MD simulation with all the restraints removed and those shown in even numbered panels are calculated from the REMD room temperature replica with restraints enforced. The distributions are normalized to 1.
